# P-29. Recombinant Zoster Vaccine Uptake Among Immunocompromised US Adults

**DOI:** 10.1093/ofid/ofae631.236

**Published:** 2025-01-29

**Authors:** Justin Gatwood, Catherine B McGuiness, Marie Yasuda, Chi-Chang Chen, Nikita Stempniewicz

**Affiliations:** GSK, Philadelphia, Pennsylvania; IQVIA, Plymouth Meeting, Pennsylvania; IQVIA, Plymouth Meeting, Pennsylvania; IQVIA, Plymouth Meeting, Pennsylvania; GSK, Philadelphia, Pennsylvania

## Abstract

**Background:**

In January 2022, ACIP recommended 2 doses of recombinant zoster vaccine (RZV) for herpes zoster (HZ) prevention in adults ≥ 19 years who are or will be immunodeficient or immunosuppressed because of disease or therapy. While this decision served to address an unmet medical need, its impact on vaccination is not fully understood as RZV uptake and series completion estimates among immunocompromised (IC) adults are lacking.

**Figure 1.**
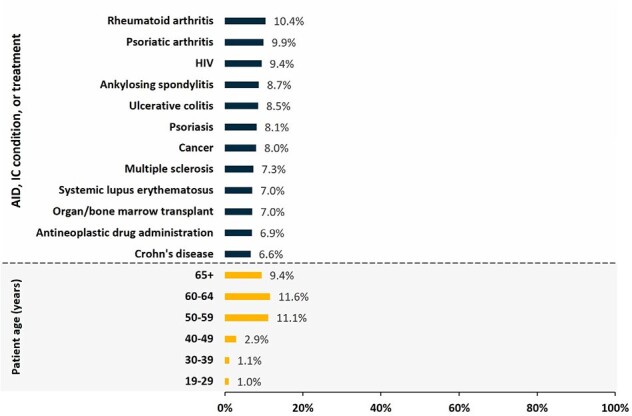
RZV Uptake (>1 dose) by Patient Age and Autoimmune Disease, Immunocompromising Condition, or Treatment Abbreviations: AID: autoimmune diseases, IC: immunocompromised, HIV: human immunodeficiency viruses, RZV: recombinant zoster vaccine.

**Methods:**

This retrospective cross-sectional analysis used IQVIA open-source medical and pharmacy claims (10/20/2021 to 06/30/2023) to describe RZV uptake and series completion among adults ≥ 19 years old with evidence of an autoimmune disorder (AID) or immunocompromising disease. RZV uptake, series completion, and dosing schedule compliance were assessed descriptively, and Kaplan-Meier analyses estimated time to series completion. Generalized estimating equations, controlling for patient demographics, clinical characteristics, and social determinants of health, predicted the odds of both RZV uptake and series completion.

**Figure 2.**
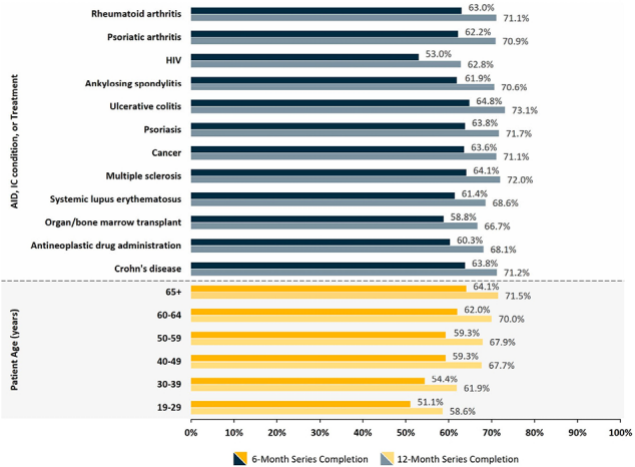
RZV Series Completion by Patient Age and Autoimmune Disease, Immunocompromising Condition, or Treatment Abbreviations: AID: autoimmune diseases, IC: immunocompromised, HIV: human immunodeficiency viruses, RZV: recombinant zoster vaccine.

**Results:**

Cumulative RZV uptake was 8.2%, increased considerably starting at age 50 years, and ranged from 6.6% to 10.4% by IC condition (Figure 1). Series completion was 62.1% (6 months post-initial dose), increasing to 70% by 12 months, with a median completion time of 4.23 months (95% confidence interval: 4.20-4.23) and 88.5% of completers complied with the recommended dosing schedule. Both series completion and dosing schedule compliance varied by age and IC condition (Figures 2 & 3). The odds of RZV uptake varied by AID/IC condition and were higher among females, those with higher incomes, minorities, and adults with more years of education. Few differences in the odds of RZV series completion by IC condition were observed; however, the odds were lower among younger and Black or Hispanic patients but increased among those with higher household incomes (Figure 4).

**Figure 3.**
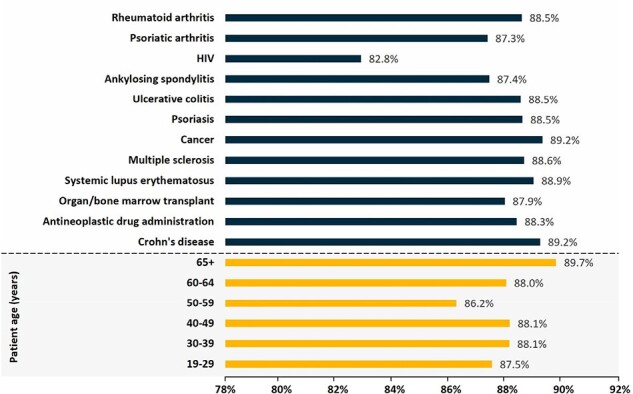
RZV Series Dosing Schedule Compliance by Age and Autoimmune Disease, Immunocompromising Condition, or Treatment Numbers reflect the proportion of series completers (2 doses) that did so within 6 months of the initial dose. Abbreviations: AID: autoimmune diseases, IC: immunocompromised, HIV: human immunodeficiency viruses, RZV: recombinant zoster vaccine.

**Conclusion:**

RZV uptake among the adult IC population in the United States is suboptimal despite ACIP guidance, yet series completion is likely among those initially vaccinated. Given the elevated risk of HZ in these patients, concerted efforts across the care team are needed to address an ongoing unmet medical need.

Funding: GSK (VEO-000679)

**Figure 4.**
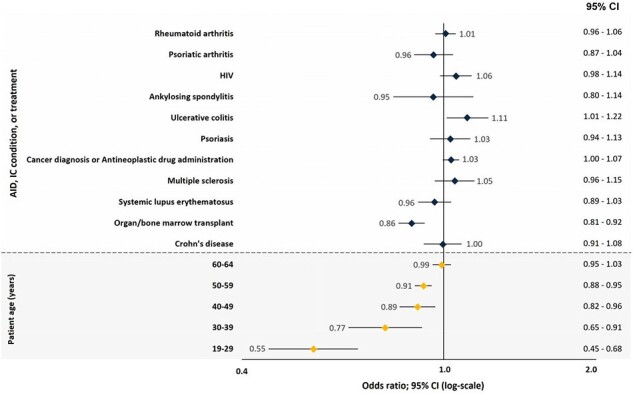
Odds of RZV Uptake among Immunocompromised Adults Abbreviations: CI: confidence interval.

**Disclosures:**

**Justin Gatwood, PhD, MPH**, AstraZeneca: Grant/Research Support|Genentech: Advisor/Consultant|GSK: Employment|GSK: Stocks/Bonds (Public Company)|Merck & Co.: Advisor/Consultant|Merck & Co.: Grant/Research Support **Catherine B. McGuiness, MA, MS**, GSK: Advisor/Consultant **Marie Yasuda, PharmD, MS**, Amgen: Grant/Research Support|Bayer: Grant/Research Support|BMS: Grant/Research Support|GSK: Advisor/Consultant|Novartis: Grant/Research Support|Sandoz: Grant/Research Support|Servier: Grant/Research Support **Chi-Chang Chen, PhD, MSPharm**, Amgen: Grant/Research Support|Bayer: Grant/Research Support|BMS: Grant/Research Support|GSK: Advisor/Consultant|Novartis: Grant/Research Support|Regeneron: Grant/Research Support **Nikita Stempniewicz, MSc**, GSK: Employment|GSK: Stocks/Bonds (Public Company)

